# Efficient AgInGaS-based QLEDs and full-color displays via uniform silver vacancy distribution

**DOI:** 10.1126/sciadv.aea0753

**Published:** 2026-02-18

**Authors:** Tianchen Li, Yuchen Yue, Hui Li, Ning Guo, Fengmian Li, Hanfei Gao, Lei Jiang, Yuchen Wu

**Affiliations:** ^1^ Key Laboratory of Bio-inspired Materials and Interfacial Science, Technical Institute of Physics and Chemistry, Chinese Academy of Sciences, Beijing 100190, P. R. China.; ^2^ College of Chemistry, Jilin University, Changchun, 130012, P. R. China.; ^3^ State Key Laboratory of Bioinspired Interfacial Materials Science, Suzhou Institute for Advanced Research, University of Science and Technology of China, Suzhou, Jiangsu 215123, P. R. China.; ^4^ Key Laboratory of Bio-Inspired Smart Interfacial Science and Technology of Ministry of Education, School of Chemistry, Beihang University, Beijing 100191, P. R. China.; ^5^ School of Future Technology, University of Chinese Academy of Sciences (UCAS), Beijing 100049, P. R. China.

## Abstract

AgInGaS (AIGS) quantum dots (QDs) are promising for displays due to their narrow full width at half maximum (FWHM) and tunable emission. However, nonuniform silver vacancy (*V*_Ag_) distribution causes emission broadening and hinders device performance improvement. Here, we present a multistep temperature control strategy that precisely regulates reaction temperature to control nucleation, cation exchange, and defect reconstruction, thereby enabling uniform *V*_Ag_ distribution in AIGS QDs. Simultaneously, we construct a dual-layer shell structure (AgGaS_2_/GaS_x_), which efficiently passivates surface defects. The synthesized red, green, and blue AIGS QDs achieve photoluminescence quantum yields (92.6, 98.5, and 53.3%) and narrow FWHMs (32, 29, and 21 nm). On the basis of these materials, we fabricated red, green, and blue QD light-emitting diodes that demonstrate external quantum efficiencies of 13.2, 8.0, and 2.9%. Moreover, the interfacial confinement self-assembly strategy enables the fabrication of full-color QD pixel arrays with resolutions up to 2032 pixels per inch, further highlighting the potential of AIGS QDs for near-eye displays.

## Teaser

Multistep temperature control enables uniform VAg distribution in AIGS QDs for efficient QLEDs and full-color displays.

## INTRODUCTION

Quantum dots (QDs) offer unique advantages for near-eye display applications due to their precisely tunable emission wavelengths and excellent solution processability, meeting the stringent requirements for high brightness, high color saturation, and wide color gamut. To date, red and green cadmium (Cd)–based QD light–emitting diodes (QLEDs) have achieved performance levels suitable for commercial display applications, while blue Cd–based QLEDs are also rapidly advancing toward commercialization (*1*–*10*). However, the inherent toxicity of Cd severely limits its widespread use in consumer electronics, driving intensive research into environmentally friendly, Cd-free alternatives. Among them, indium phosphide (InP)–based QDs, owing to their tunable electronic structure and favorable environmental compatibility, are considered one of the most promising heavy metal–free emissive materials (*11*–*15*). Although the external quantum efficiencies (EQEs) of red and green InP QLEDs have approached those of Cd-based devices, their broad emission spectra and yellow-green emission limit effective coverage of the ITU-R Rec. 2020 color gamut. Therefore, the development of heavy metal–free QDs that simultaneously exhibit narrowband emission and wide color gamut coverage remains a critical bottleneck for next-generation high-end display technologies.

Recently, AgInGaS (AIGS) QDs have attracted considerable attention due to their narrower full width at half maximum (FWHM) compared to InP-based materials. Although strategies such as core-shell structural design and surface defect passivation have been used to enhance their photoluminescence quantum yield (PLQY) and device performance, AIGS QDs still face challenges of limited color purity, incomplete color gamut coverage, and suboptimal QLED efficiencies. While Lee *et al.* (*16*) have confirmed that one of the main origins of broad emission is the surface states of AIGS QDs and made important contributions to shell passivation, the reported red, green, and blue AIGS QDs still exhibit FWHMs of approximately 42, 30, and 34 nm, respectively. In addition, the AIGS-based QLED devices show relatively low EQEs of 1.8, 5.4, and 0.5% (*17*–*24*), which remain substantially lower than those achieved with Cd- or InP-based QDs. Previous studies have also demonstrated that the luminescence of AIGS QDs predominantly originates from defect-mediated band-edge recombination, particularly the transition of conduction band electrons to hole states associated with silver vacancies (*V*_Ag_) (*24*, *25*). As the dominant acceptor-type defect, inhomogeneous *V*_Ag_ distribution induces localized state fluctuations and band-edge broadening, leading to spectral broadening, reduced radiative efficiency, and ultimately degraded device performance. Therefore, achieving spatially uniform control of *V*_Ag_ has emerged as another key scientific challenge for improving the optical quality of AIGS QDs and advancing their application in high-performance QLEDs.

Here, we developed a multistep temperature control strategy for the synthesis of AIGS QDs, in which precise control over reaction temperature and heating rate enables the sequential occurrence of key processes, including nucleation, cation exchange, and defect reconstruction across distinct timescales. This approach allows for the precise regulation of the spatial distribution of *V*_Ag_ within the QDs. In parallel, we construct a dual-layer shell structure consisting of AgGaS_2_ (AGS) and GaS_x_ (GS), which effectively passivates surface defect states, thereby further enhancing the optical quality of the material. As a result of this strategy, the synthesized red, green, and blue AIGS QDs exhibit high PLQYs of 92.6, 98.5, and 53.3%, with emission peaks centered at 631, 513, and 446 nm, and narrow FWHMs of 32, 29, and 21 nm, respectively. These results notably outperform previously reported AIGS QDs. On the basis of these high-quality materials, we fabricated red, green, and blue QLEDs that achieve EQEs of 13.2, 8.0, and 2.9%, respectively. Moreover, by integrating an interfacial confinement self-assembly strategy, we successfully realized high-density, highly uniform QD pixel arrays, enabling the fabrication of a full-color external excited pattern with an ultrahigh resolution of 2032 pixels per inch (PPI). These results not only highlight the distinct advantages of the multistep temperature control strategy and dual-layer shell structure in structural and defect engineering but also underscore the potential of AIGS QDs for use in next-generation, high-resolution, wide color gamut, and environmentally friendly display technologies.

## RESULTS

### Multistep temperature control strategy for uniform *V*_Ag_ distribution

**Fig. 1.**
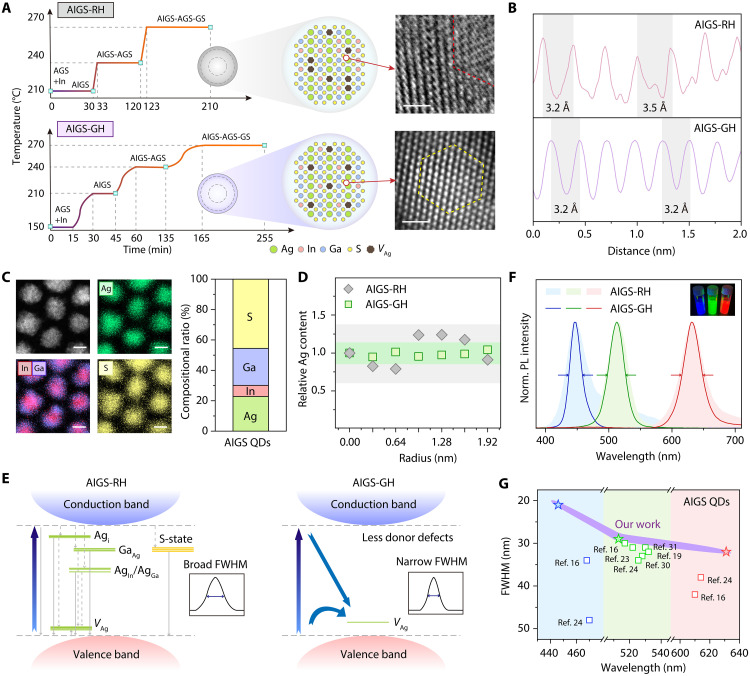
High-quality AIGS QDs via uniform *V*_Ag_ distribution. (**A**) Structural models and aberration-corrected TEM images of individual AIGS-RH QD and AIGS-GH QD (scale bar, 1 nm). (**B**) Atomic intensity profiles along the (110) planes of individual AIGS-RH QD and AIGS-GH QD. (**C**) Energy-dispersive x-ray spectroscopy elemental mapping (left) and element distribution (right) of AIGS-GH QDs (scale bars, 5 nm). (**D**) Relative atomic content of Ag in AIGS-RH and AIGS-GH QDs as a function of etching depth. (**E**) Schematic illustration of electronic band structure of AIGS-RH and AIGS-GH QDs and their different paths of recombination. (**F**) PL spectra of red, green, and blue AIGS-GH QDs (inset: a photographic image of red, green, and blue AIGS-GH QDs). (**G**) Reported narrow FWHM versus PL wavelength of state-of-the-art AIGS QDs (*16*, *19*, *23*, *24*, *30*, *31*).

To synthesize high-quality AIGS QDs, we developed a multistep temperature control strategy, as illustrated in fig. S1. In AIGS QDs, to ensure high PLQY, the input Ag precursor is usually kept substoichiometric, which means that a certain density of uniform *V*_Ag_ is crucial to get high-quality QDs. While during conventional rapid heating method (AIGS-RH), lattice dislocations are prone to form in AIGS QDs. When the temperature increases too quickly, Ag^+^ ions migrate rapidly between lattice sites due to the high mobility (*26*), which means that *V*_Ag_ tend to distribute unevenly across the lattice, broadening the distribution of Ag-related defect states and ultimately increasing the FWHM of the PL spectrum. In contrast, by using a gradual heating strategy (AIGS-GH), the migration rate of Ag^+^ ions are effectively reduced, thereby minimizing lattice disturbances and enabling the formation of defect-free AIGS QDs, as illustrated in Fig. 1A. Structural characterization revealed a clear difference in crystalline quality between AIGS-RH and AIGS-GH QDs. Spherical aberration-corrected transmission electron microscopy (TEM) images show that AIGS-RH QDs contained obvious lattice defects, including stacking faults with expanded lattice spacing (~3.5 Å). In contrast, AIGS-GH QDs display uniform lattice spacing (~3.2 Å) and high crystallinity (Fig. 1B), supported by energy-dispersive x-ray spectroscopy mapping and atomic composition analysis (Ag: 22%, In: 7%, Ga: 25%, S: 46%) (Fig. 1C). X-ray photoelectron spectroscopy (XPS) depth profiling (Fig. 1D) reveals that AIGS-RH QDs exhibit a relatively nonuniform Ag distribution, indicating pronounced radial inhomogeneity. In contrast, AIGS-GH QDs show an almost homogeneous Ag distribution along the radial direction. Moreover, Ag K-edge extended x-ray absorption fine structure (EXAFS) analyses (fig. S2) provide further direct evidence. In the R-space spectra (fig. S2A), both AIGS-GH and AIGS-RH exhibit a distinct first-shell Ag-S peak. Fitting of this peak yields coordination numbers of 3.59 for AIGS-GH and 3.66 for AIGS-RH, both below the ideal tetrahedral value of 4, thereby directly confirming the ubiquitous presence of *V*_Ag_ in both samples. In addition, the Debye-Waller factor is slightly lower for AIGS-GH (0.0096) than for AIGS-RH (0.0102), indicating reduced local structural disorder. In k-space (fig. S2B), the oscillations of AIGS-GH remain more coherent across the full wave number range, consistent with smaller structural fluctuations. In the wavelet transform contour plots (fig. S2, C and D), AIGS-GH exhibits compact, well-localized Ag-S (~2 Å) and a clear high-k Ag-Ag (~3 Å) lobe, meaning lower static disorder. By contrast, AIGS-RH shows a broadened Ag-S lobe and a weakened, diffuse high-k Ag-Ag feature (*27*, *28*). Collectively, these results provide compelling structural evidence that *V*_Ag_ are more uniformly distributed in AIGS-GH than in AIGS-RH. The uniform distribution of *V*_Ag_ effectively suppresses defect-related donor-acceptor pair (DAP) recombination, shifting the emission mechanism toward band-edge transitions involving free carriers localized at *V*_Ag_ sites (Fig. 1E), thereby reflecting improved structural and electronic homogeneity.

Uniform *V*_Ag_ distribution directly translate into superior optical performance. The resulting AIGS-GH QDs exhibited high-purity red (631 nm, FWHM: 32 nm), green (513 nm, FWHM: 29 nm), and blue (446 nm, FWHM: 21 nm) luminescence (Fig. 1F). Compared with AIGS-RH QDs, AIGS-GH QDs display narrower emission peaks, as further corroborated by PL spectra both samples at different synthesis stages (fig. S3). Notably, relative to conventional InP QDs, AIGS-GH achieves markedly color purity (*11*, *12*, *29*), underscoring the advantage of this material system. For the blue-emissive AIGS-GH QDs, a noticeable red-side tail is still observed. This spectral asymmetry mainly arises from compositional inhomogeneity, especially *V*_Ag_ within the QDs. The photographic images of color evolution during synthesis are displayed in fig. S4. TEM images of AIGS-GH QDs with different emission colors are shown in fig. S5, revealing average particle sizes of 11.43 nm (red), 9.13 nm (green), and 7.26 nm (blue). Impressively, this approach yielded the narrowest reported emission linewidths reported for AIGS QDs to date (Fig. 1G and table S1) (*16*, *19*, *23*, *24*, *30*, *31*).

### Dual-layer shell structure for effectively passivating surface defects

**Fig. 2.**
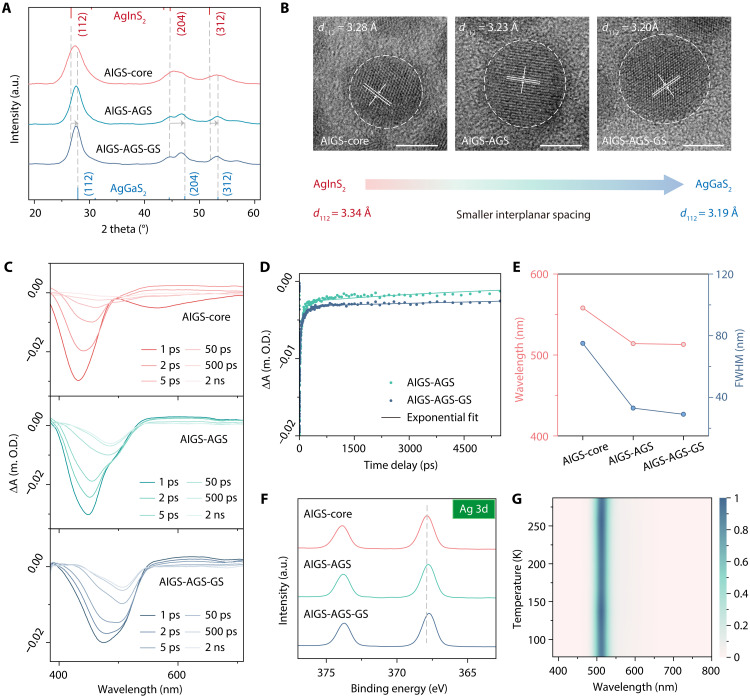
Characterization of changes during the dual-layer shell coating process of AIGS QDs. (**A**) XRD patterns and (**B**) high-resolution TEM (HRTEM) images of AIGS-core, AIGS-AGS, and AIGS-AGS-GS QDs (scale bars, 5 nm). (**C**) The fs-TA spectra of AIGS-core, AIGS-AGS, and AIGS-AGS-GS QDs. (**D**) The kinetic traces of ground-state bleaching maximum of AIGS-AGS and AIGS-AGS-GS QDs. (**E**) PL peak wavelength and FWHM of green AIGS-core, AIGS-AGS, and AIGS-AGS-GS QDs. (**F**) High-resolution Ag XPS spectra of AIGS-core, AIGS-AGS, and AIGS-AGS-GS QDs. (**G**) Two-dimensional temperature-dependent PL spectra for green AIGS-AGS-GS QDs from 77 to 287 K. a.u., arbitrary unit.

To further demonstrate the enhancement of optical properties in AIGS QDs achieved by the dual-layer shell structure, we conducted a series of characterizations of changes during the dual-layer shell coating process of green AIGS QDs (AIGS-core, AIGS-AGS, and AIGS-AGS-GS) synthesized via the multistep temperature control strategy. We systematically investigated the evolution of x-ray diffraction (XRD) patterns and surface morphologies at different stages, including AIGS-core, AIGS-AGS, and AIGS-AGS-GS QDs. As shown in Fig. 2A, the XRD pattern of AIGS-core closely matches the standard diffraction peaks of tetragonal AgInS_2_. Following GH shelling process, the XRD peaks of AIGS-AGS QDs shift slightly toward those characteristics of tetragonal AgGaS_2_. The XRD pattern of AIGS-AGS-GS QDs aligns even more closely with that of tetragonal AgGaS_2_, while distinct diffraction features of AgInS_2_ persist. In addition, high-resolution TEM images (Fig. 2B) reveal increasingly uniform particle morphology and improved structural order. The QDs progressively exhibit more regular structures as the AGS and GS coatings are applied, accompanied by a gradual decrease in lattice spacings. The measured lattice spacings are 3.28 Å for the AIGS-core, 3.25 Å for AIGS-AGS QDs, and 3.20 Å for AIGS-AGS-GS QDs. For comparison, the lattice spacings of AgInS_2_ and AgGaS_2_ are 3.34 and 3.19 Å, respectively. These results indicate a surface structural transition from AgInS_2_-dominant to AgGaS_2_-dominant.

To further probe the optoelectronic impact of this dual-layer shelling structure, we performed transient absorption (TA) spectroscopy to investigate exciton dynamics in AIGS-core, AIGS-AGS, and AIGS-AGS-GS QDs (Fig. 2C; pseudo-color TA maps of three different color AIGS-AGS-GS QDs are shown in fig. S6). All samples exhibit two distinct bleaching peaks. The ground-state bleaching recovery dynamics (~473 nm for AIGS-AGS, ~480 nm for AIGS-AGS-GS) were analyzed by fitting the decay curves using a triexponential function (Fig. 2D and table S2). The fitting results reveal that exciton recombination is primarily dominated by a “free-to-bound” mechanism. The progression of PL peak positions and FWHMs for green AIGS-core, AIGS-AGS, and AIGS-AGS-GS QDs is also summarized in Fig. 2E. The AIGS-core exhibits a broad FWHM (72 nm), indicative of pronounced surface defects and multiple defect-related energy states leading to complex DAP recombination (*25*). As AGS shell is coated, surface defects are effectively passivated to some degree. The AIGS-AGS exhibits a FWHM of 33 nm. After GS shell is also coated, surface defects are more passivated effectively, resulting in narrower FWHM (29 nm). The corresponding absorption and PL spectra, together with the progression of peak wavelength and FWHM values for red, green, and blue AIGS-core, AIGS-AGS, and AIGS-AGS-GS QDs for both RH and GH conditions, are presented in fig. S7. These comparisons clearly highlight that the multistep temperature control strategy together with dual-layer shell structure yields narrower emission linewidths and improved optical uniformity. In addition, XPS measurements were conducted (full spectra shown in fig. S8 and high-resolution Ag, In, Ga, and S spectra in fig. S9). We paid special attention to the Ag XPS spectra (Fig. 2F) across the three types of QDs. The Ag 3d peaks exhibit a systematic shift toward higher binding energy. This trend can be attributed not only to the controlled formation and uniform spatial distribution of *V*_Ag_ induced by the gradient-heating dual-layer shelling process but also to the gradual compositional evolution from higher In content to higher Ga content within the AIGS alloy lattice. Since Ga is less metallic than In, the increasing Ga fraction places Ag atoms in a more oxidative environment, further contributing to the observed positive shift. Moreover, temperature-dependent PL measurements (Fig. 2G) confirm the excellent optical stability of AIGS-AGS-GS QDs, with negligible changes in both emission intensity over time. Negligible peak-energy shifts may be dominated by localized states, such as donor-acceptor or free-to-bound transitions (*31*). The fluorescence lifetimes of red, green, and blue QDs at different stages (AIGS-core, AIGS-AGS single-shell, and AIGS-AGS-GS dual-layer shell), measured by time-resolved PL spectroscopy, are summarized in fig. S10 and table S3. The carrier lifetime of AIGS-AGS QDs is markedly reduced, indicating suppression of nonradiative recombination and partial passivation of surface defects, which simultaneously leads to a substantial increase in PLQY. AIGS-AGS-GS QDs further enhance defect passivation and carrier confinement, yielding an additional PLQY improvement and enhanced spectral stability. Collectively, these findings highlight that the dual-layer shell structure effectively passivates surface defects, and it could facilitate improved QLED performance.

### Performance characterization of AIGS-based QLEDs

**Fig. 3.**
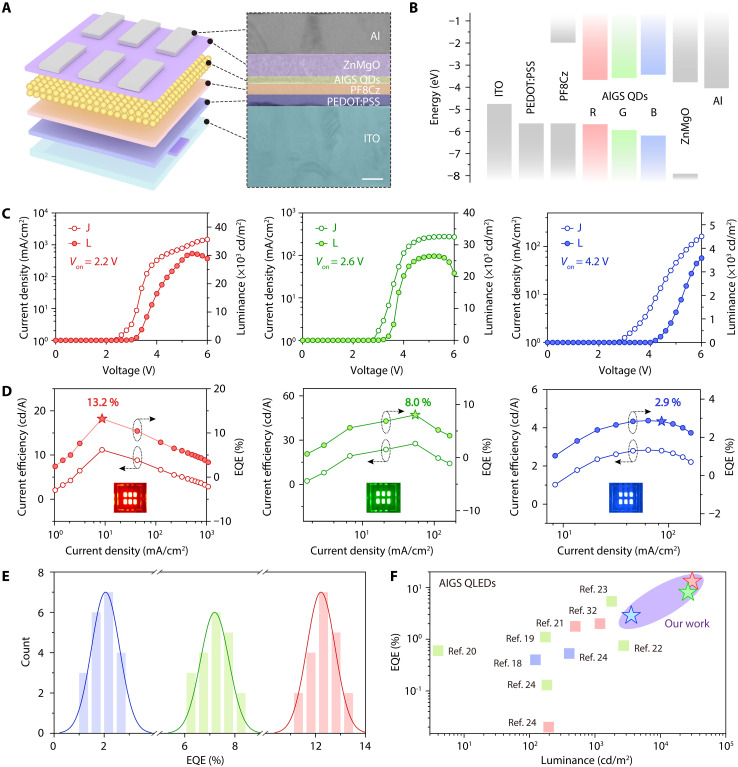
The performance of AIGS-based QLEDs. (**A**) Schematic illustration (left) and cross-sectional HRTEM image (right) of the QLED device structure (scale bar, 50 nm). (**B**) Energy level alignment diagram of the various functional layers. (**C**) Current densities (hollow circles) and luminance (solid circles) versus voltage and (**D**) current efficiencies (hollow circles) and EQEs (solid circles) versus current densities of red, green, and blue AIGS-based QLEDs. (**E**) Histogram of peak EQEs measured from 20 devices for each color. (**F**) Reported peak EQE versus peak luminance of AIGS-based QLEDs (*18*–*24*, *32*).

Encouraged by the improved homogeneity and superior structural stability of AIGS QDs, we fabricated QLEDs using the synthesized high color-purity red, green, and blue AIGS QDs using multistep temperature control strategy with dual-layer shell structure. The device architecture was composed of indium tin oxide (ITO)/poly(3,4-ethylenedioxythiophene):poly(styrenesulfonate) (PEDOT:PSS)/poly[(9,9-dioctylfluorenyl-2,7-diyl)-alt-(9-(2-ethylhexyl)carbazole-3,6-diyl)] (PF8Cz)/AIGS QDs/ZnMgO/Al. A cross-sectional TEM image of an individual AIGS QLED (Fig. 3A) clearly reveals the sequential stacking of the functional layers and provides detailed information on the thickness of each layer. The corresponding energy band structures for the red, green, and blue QLEDs are depicted in Fig. 3B. The optical bandgaps (E_g_) of the QDs were determined by extrapolation of the absorption spectra, as shown in fig. S11, and the ultraviolet (UV) photoelectron spectroscopy results are provided in fig. S12.

The current density–voltage-luminance (*J-V-L*) characteristics and current efficiency–current density–EQE curves for devices based on red, green, and blue AIGS QDs are shown in Fig. 3 (C and D). The devices exhibited impressive peak luminance of 30,670 cd m^−2^ (red), 26,450 cd m^−2^ (green), and 3580 cd m^−2^ (blue), with corresponding peak EQEs of 13.2, 8.0 and 2.9%, respectively. For a comprehensive evaluation of device performance, the angular luminance distributions of red, green, and blue AIGS-based QLEDs are presented in fig. S13. At an initial luminance of 4600, 3620, and 910 cd m^−2^, the *T*_95_ lifetimes of red, green, and blue AIGS-GH-based QLEDs are 1.50, 0.98, and 0.52 hours, respectively. By extrapolating these *T*_95_ lifetimes to an initial luminance of 100 cd m^−2^ using the relation *L*_0_*^n^T*_95_ = const, we obtain estimated lifetimes of approximately 1129, 543, and 28 hours for the red, green, and blue devices, respectively, as shown in fig. S14. Here, *L*_0_ denotes the initial luminance, *T*_95_ represents the corresponding life, and *n* is the acceleration factor, with values of 1.73, 1.76, and 1.81, respectively. These enhanced performance metrics are primarily attributed to the improved uniformity in *V*_Ag_, which effectively suppresses nonradiative recombination and improves carrier injection balance. To further substantiate this conclusion, we fabricated and tested devices based on AIGS-RH QDs. In these cases, the electroluminescence characteristics, including maximum luminance and peak EQE, are notably inferior compared to the devices presented in this work (fig. S15). This direct comparison strongly supports the critical role of uniform *V*_Ag_ distribution in enabling the observed high performance. The stability and reproducibility of the device performance were further verified by testing 20 different red, green, and blue AIGS QLEDs, as shown in Fig. 3E. Moreover, Fig. 3F highlights that the performance of these AIGS QLEDs outperforms that of previously reported AIGS QLEDs (see details in table S4) (*17*–*24*, *32*). Compared to InP-based QLEDs, the AIGS-based QLEDs exhibit a broader color gamut (fig. S16).

### Externally excited full-color pattern enabled by AIGS QDs

**Fig. 4.**
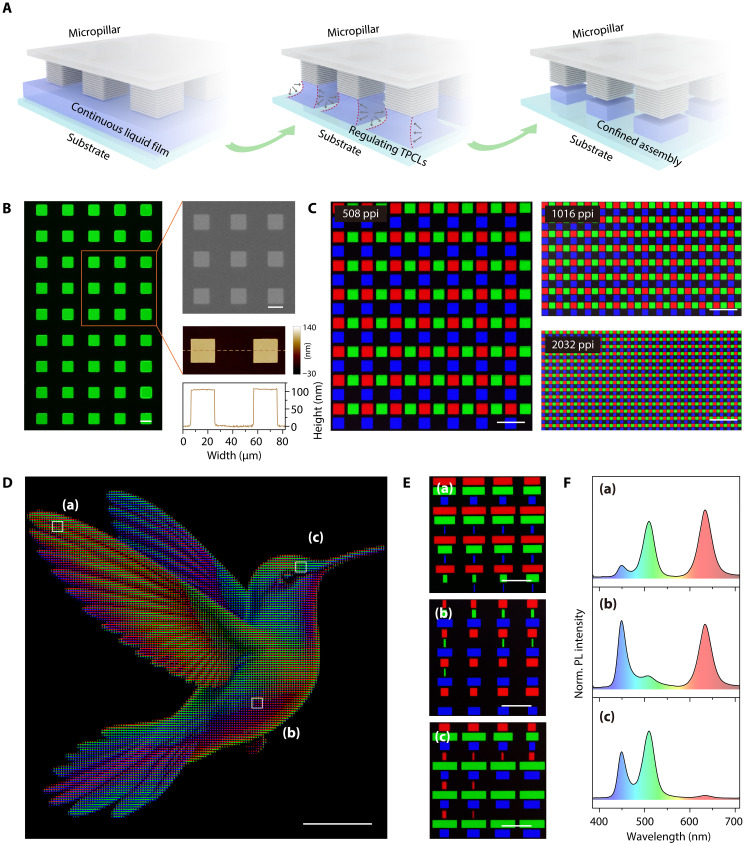
Externally excited full-color pattern based on AIGS QDs. (**A**) Schematic illustrations of the confined assembly process with controlling TPCL’s behaviors. (**B**) Fluorescence microscope image (left), SEM image (top right), and AFM image (bottom right) of green QD microarrays (scale bars, 20 μm). (**C**) Fluorescence microscope image of full-color QD microarrays (scale bars, 20 μm). (**D**) PL image of a full-color image (scale bar, 3 mm). (**E**) Magnified fluorescent images showing the scaled pixelated RGB patterns from the areas indicated in (D) (scale bars, 100 μm). (**F**) PL spectra of the areas indicated in (D).

To demonstrate the application potential of AIGS QDs in display technologies, we developed an interfacial confinement self-assembly strategy to achieve high-quality and high-resolution square arrays. This approach used micropillar templates with asymmetric wettability, hydrophilic tops, and hydrophobic sidewalls (as detailed in fig. S17)—to precisely modulate the dynamic behavior of QD solutions at the three-phase contact lines (TPCLs). As illustrated in Fig. 4A, during solvent evaporation, capillary bridges spontaneously formed within the confined space between the template and the substrate, while TPCLs on the micropillar tops were pinned because of abrupt changes in surface wettability and geometry, forming coaxial gas-liquid interfaces that confined the QD assembly. Continuous solvent evaporation induced sliding of the TPCLs on the substrate side, and this asymmetric motion enhanced inward Marangoni flows, suppressing the coffee-ring effect and promoting highly ordered QD deposition. The entire confined assembly process is illustrated in fig. S18. The resulting green AIGS QD microarrays (Fig. 4B), as confirmed by fluorescence microscopy, scanning electron microscopy (SEM), and atomic force microscopy (AFM), exhibited sharply defined edges and high surface uniformity (corresponding surface roughness is provided in fig. S19). Red and blue microarrays fabricated similarly are shown in fig. S20.

To realize full-color externally excited pattern, sequential assembly of red, green, and blue microarrays on a single substrate was required. However, subsequent solution deposition steps could damage previously assembled microarrays. To overcome this challenge, we used an in situ ligand exchange process, replacing oleyamine (OAm) ligands with 3-mercaptopropionic acid (MPA), thereby enhancing structural robustness without compromising morphological integrity. As shown in fig. S21A, this process substantially improves wettability, with the contact angle decreasing from 17.3° to 4.8°. Furthermore, Fourier transform infrared analysis (fig. S21B) confirms the ligand exchange. Before exchange, strong absorption peaks at 2921 and 2851 cm^−1^ are attributed to C-H_x_ vibrations of the long alkyl chains from OAm ligands. After exchange, distinct peak emerges at 1703 cm^−1^, corresponding to the C=O vibration of MPA, evidencing the successful substitution of a substantial fraction of OAm ligands. As a result, well-ordered micropatterns are obtained, as further demonstrated by the fluorescence microscopy images in fig. S22. High-resolution full-color AIGS QD microarrays with 508, 1016, and 2032 PPI are shown in Fig. 4C. To demonstrate full-color rendering capability, the relative red, green and blue (RGB) brightness values were translated into proportional lengths of microrectangles, enabling the design of tunable color microarrays based on RGB value decomposition (figs. S23 and S24). Using specially designed micropillar templates, photoluminescent panels with customizable patterns were assembled via this interfacial confinement self-assembly strategy. The final full-color externally excited pattern is shown in Fig. 4D, with zoomed-in microarrays in Fig. 4E and the corresponding PL spectra in Fig. 4F. Statistical analysis of the QD microarray pattern width compared to the pillar width is shown in fig. S25. These results underscore the tremendous promise of AIGS QDs in advanced display applications, enabling high-definition, full-color, solution-processable emissive panels.

## DISCUSSION

In this study, we successfully used a multistep temperature control strategy and dual-layer shell structure to synthesize high color-purity AIGS QDs with record-narrow FWHMs of 32 nm (red), 29 nm (green), and 21 nm (blue). Detailed analyses reveal that uniform distribution of *V*_Ag_ is the key to narrowing emission linewidths and improving radiative recombination via free-to-bound transitions. Using these optimized QDs, we achieved high-performance red, green, and blue QLEDs with luminance values up to 30,670, 26,450, and 3580 cd m^−2^ and peak EQEs of 13.2, 8.0, and 2.9%. In addition, we developed an interfacial confinement self-assembly strategy for fabricating full-color externally excited pattern with resolutions up to 2032 PPI. Together, these advancements establish AIGS QDs as promising candidates for next-generation, Cd-free near-eye display technologies.

## MATERIALS AND METHODS

### Materials

Silver (I) iodide (AgI, 99.999%), indium (III) acetate [In(ac)_3_, 99.99%], *n*-trioctylphosphine (99%), and 1-octadecene (ODE, 90%) were purchased from Alfa. Sulfur powder (S, 99.9%), oleic acid (OA, 90%), myristic acid (HMy, 98%), gallium (III) acetylacetonate [Ga(acac)_3_, 99.99%], OAm (70%), 1-dodecanethiol (DDT, ≥98%), chlorobenzene, acetone, and isopropyl alcohol were purchased from Sigma-Aldrich. Gallium chloride (GaCl_3_, 98%) was purchased from Aladdin. PEDOT:PSS (AI 4083) was purchased from Heraeus Deutschland GmbH & Co. KG. PF8Cz was purchased from Volt-Amp Optoelectronics Tech. Co. Ltd., Dongguan, China. Toluene (anhydrous, ≥99.5%), ethanol (anhydrous, ≥99.5%), *n*-hexane (anhydrous, 99.9%), and *n*-octane (anhydrous, 99%) were purchased from Sinopharm Chemical Reagent Co. Ltd. All chemicals, unless otherwise stated, were used as received.

### Preparation of precursor

All chemistry is conducted with Schlenk line technique. Gallium oleate (0.5 M) [Ga(OA)_3_] and 0.5 M indium myristate [In(My)_3_] stock solutions in ODE are prepared for cation precursors, and S dissolved in OAm (S-OAm) is prepared for anion precursors. For Ga(OA)_3_ preparation, 10 mmol of Ga(acac)_3_ and 30 mmol of OA are mixed in a three-neck flask (250 ml), degassed at 130°C for 6 hours, backfilled with N_2_, and diluted to 0.5 M concentration with ODE. For In(My)_3_ preparation, 10 mmol of In(OA)_3_ and 30 mmol of HMy are mixed, degassed at 120°C for 6 hours, backfilled with N_2_, and diluted to 0.5 M concentration with ODE. For S-OAm stock solution, 10 mmol of sulfur powder is mixed in 20 ml of OAm, degassed at 50°C for 2 hours, and backfilled with N_2_.

### Preparation of Ag-S-Ga(OA)_2_

All synthesis was carried out under N_2_ atmosphere through the Schlenk line technique. For preparing Ag_2_S nanoparticles, 1 mmol of AgI and 5 ml of OAm were loaded in a three-neck flask and degassed at 50°C for 1 hour. After the flask was backfilled with N_2_, 2.5 ml of DDT and 1 ml of 0.5 M S-OAm were injected into the reaction flask to form Ag_2_S NPs. To transform Ag_2_S NPs to Ag-S-Ga(OA)_2_, 4.5 ml of 0.5 M Ga(OA)_3_ and 8 ml of 0.5 M S-OAm were added into the reaction flask and the temperature was elevated to 210°C for 0.5 hours.

### AIGS-GH QDs synthesis

For the synthesis of AIGS cores with different emission colors, the amounts of In(ac)_3_ and HMy were adjusted accordingly: 0.5 and 1.5 mmol for red, 0.08 and 0.24 mmol for green, and 0.01 and 0.03 mmol for blue. In a typical synthesis (green cores), a reaction flask containing 0.08 mmol of In(ac)_3_, 0.24 mmol of HMy, 3.5 ml of ODE, and 15 ml of OAm was degassed at 110°C for 1 hour, backfilled with N_2_, and heated to 150°C. Then, 4.5 ml of Ag-S-Ga(OA)_2_ stock solution were swiftly injected into the flask to initiate nucleation and subsequent growth. The reaction temperature was maintained for 15 min and then gradually increased to 210°C at 4°C min^−1^ and held for 15 min to complete nucleation. For the AGS shell growth, the temperature was gradually raised to 240°C at 2°C min^−1^, while a mixture of 1 ml Ag-S-Ga(OA)_2_, 0.5 ml0.5 M S-OAm, and 0.1 ml 5 M GaCl_3_ (in ethanol) was injected at 1.6 ml hour^−1^, and the reaction temperature was maintained for 1.5 hours. Subsequently, for the GS shell growth, the temperature was further gradually increased to 270°C at 1°C min^−1^, while a mixture of 1 ml Ga(OA)_3_, 1.5 ml S-OAm, and 0.1 ml GaCl_3_ solution was injected at 2.5 ml hour^−1^, and the reaction temperature was maintained for 2 hours. Last, the reaction was quenched by water-bath cooling to room temperature, and the resulting AIGS-RH QDs were purified twice by ethanol precipitation/redispersion in toluene, followed by final dispersion in toluene/hexane/octane for further characterization and applications.

### Fabrication of QLEDs

ITO-coated glass substrates were used to fabricate the devices. They were cleaned in an ultrasonic bath with detergent, deionized water, acetone, and isopropanol for 15 min each, and then subjected to UV ozone sterilization for 15 min. A layer of PEDOT:PSS served as the hole injection layer and was spin-coated to the ITO substrates at a speed of 4000 rpm, followed by baking at a temperature of 150°C for 30 min in air. PF8Cz was then spin coated from a solution in chlorobenzene at a concentration of 8 mg ml^−1^ at 3000 rpm for 40 s and baked at 120°C for 30 min. Subsequently, the red, green, or blue AIGS QD solution dissolved in octane at a concentration of 20 mg ml^−1^ was spin-coated at a speed of 2000 rpm for 40 s, and then annealed at 100°C for 5 min. Subsequently, a layer of ZnMgO (for detailed description of the synthesis process of ZnMgO, see note S1 and the Supplementary Materials) was spun-coated onto the substrate at 3000 rpm and then annealed at 60°C for 30 min. Thereafter, a layer of aluminum with a thickness of 100 nm was deposited via thermal evaporation. Ultimately, the device fabrication was completed by encapsulating with UV-curable epoxy resin and cover glass in a glovebox.

### Characterization

All fluorescent images were taken by an optical microscopy (Olympus, DP80). The morphology of the QDs were evaluated by TEM (JEOL, 2100F, Japan) at 200 kV operating voltage. XRD data were acquired using an x-ray diffractometer (Bruker Nano Inc.) with monochromatized Cu Kα radiation (λ = 1.5406 Å). UV-Vis spectra were obtained by Cary 7000 (Agilent, USA). The emission spectra and PL decay curves of QDs were analyzed by the Edinburgh instrument FLS 1000. X-ray absorption spectroscopy measurements were conducted at BM23 beamline at ESRF (European Synchrotron Radiation Facility). EXAFS analyses were processed by Athena and Artemis software within the IFEFFIT package. TA measurements were performed using femtosecond laser pulses from an 800-nm laser beam (1-kHz pulse repetition rate and 25-fs pulse duration) generated by a regenerative amplified Ti:Sapphire laser system (Coherent Co.). The output of the amplifier was split into two streams of pulses with a beam splitter. Residual stream was directed into an ultrafast spectroscopic system [Helios pump-probe system (Ultrafast Systems) to generate the white light continuum probe beam (300- to 2600-nm window with various optical filters]. The pump wavelength was set at 370 nm. Delaying the probe pulse relative to the pump allowed a time window of up to 5 ns. The instrument response function was determined to be ≈200 fs using a cross-correlation procedure. The sample was filled in a 1-mm-thick quartz cell for TA experiment, which was continuously swayed to prevent the optical damage. All SEM images were tested at 10-kV and 10-μA current using the Hitachi SU8010 (Japan) instrument. AFM (Bruker Nano Inc.) was used to measure the morphology of the QD arrays.

### Characterization of QLEDs

All devices were tested within a nitrogen-filled glove box using a customized test socket. The current density-voltage-luminance (*J*-*V*-*L*) curves and EQE of the devices were measured using a commercial system (XPQY-EQE-Adv, Guangzhou Xipu Optoelectronics Technology Co. Ltd.).
