# Supplementary material for: Efficient AgInGaS-based QLEDs and full-color displays via uniform silver vacancy distribution
Source: Sci Adv. 2026 Feb 18;12(8):eaea0753. doi: 10.1126/sciadv.aea0753 (PMC12915620; doi:10.1126/sciadv.aea0753)
Supplement: Supplementary file 1 — Supplementary Text Figs. S1 to S25 Tables S1 to S4 [file sciadv.aea0753_sm.pdf]

Science Advances
AAAS

Supplementary Materials for

Efficient AgInGaS-based QLEDs and full-color displays via uniform silver vacancy distribution

Tianchen Li *et al.*

Corresponding author: Hui Li, lihui-t22031@ustc.edu.cn; Hanfei Gao, gaohanfei15@mails.ucas.ac.cn; Yuchen Wu, wuyuchen@iccas.ac.cn

Sci. Adv. **12**, eaea0753 (2026)
DOI: 10.1126/sciadv.aea0753

This PDF file includes:

Supplementary Text
Figs. S1 to S25
Tables S1 to S4

Supplementary Text

Supplementary Note S1. Synthesis of $Zn_{0.9}Mg_{0.1}O$ nanocrystals

1-1. Materials

Tetramethylammonium hydroxide pentahydrate (TMAH·$5H_2O$, 97%), zinc acetate dihydrate ($Zn(OAc)_2 \cdot 2H_2O$, 99.995%) and dimethyl sulfoxide (DMSO, 99.7%) were purchased from Aladdin. Magnesium acetate tetrahydrate ($Mg(OAc)_2 \cdot 4H_2O$, 99%) were purchased from Sigma-Aldrich.

1-2. Synthesis of $Zn_{0.9}Mg_{0.1}O$ nanocrystals

A total of 2.7 mmol of $Zn(OAc)_2 \cdot 2H_2O$ and 0.3 mmol of $Mg(OAc)_2 \cdot 4H_2O$ were dissolved in 30 mL of DMSO to form the precursor solution. Subsequently, 8.75 mL of a 0.5 mmol·mL^{-1} solution of TMAH·$5H_2O$ in ethanol was gradually added under constant stirring. The reaction mixture was maintained at 18 °C throughout the process and stirred for an additional 2 hours to facilitate nanocrystal formation. Upon completion of the reaction, the product was isolated by centrifugation and washed twice with ethanol to remove residual impurities. Finally, the resulting precipitate was redispersed in ethanol to obtain a $Zn_{0.9}Mg_{0.1}O$ nanoparticle dispersion with a concentration of 20 mg·mL^{-1}.

Supplementary Note S2. FAS modification

2-1. Materials

Heptadecafluorodecyltrimethoxysilane (FAS, 98%) and acetone (98%) were purchased from Aladdin.

2-2. FAS modification

Initially, a substrate coated with SU-8 photoresist was placed atop the microcolumns, thereby protecting the upper surfaces during subsequent processing. The upper regions of the microcolumns were preserved by carefully detaching the SU-8-coated substrate. Following this, the sidewalls and intercolumnar gaps of the microcolumn templates were functionalized to exhibit hydrophobicity through FAS treatment. For the silanization process, the top-protected microcolumn templates were positioned in a glass Petri dish, into which 20 μL of FAS solution was introduced. The samples were subjected to vapor-phase silanization under reduced pressure at ambient temperature for 24 hours, followed by a thermal post-treatment at 90 °C for 2 hours to complete the modification. Subsequently, the SU-8 photoresist was removed using acetone, yielding microcolumns with asymmetric wettability between the top surfaces and the sidewalls.

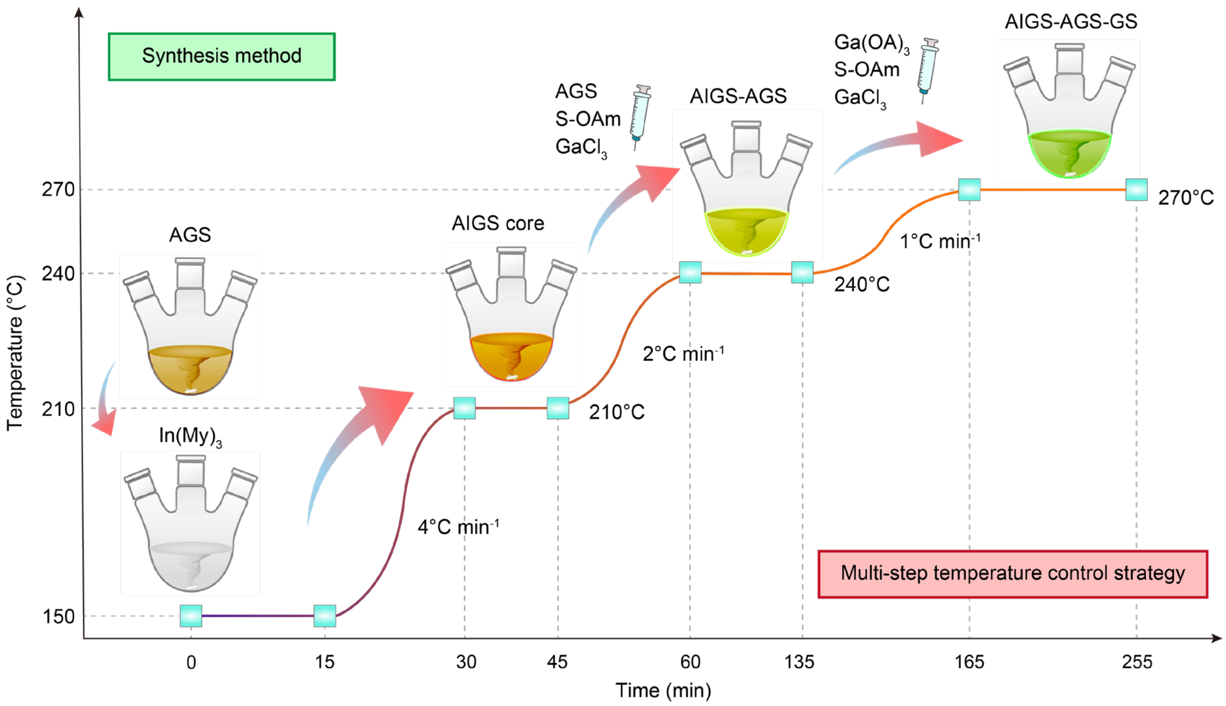

Fig. S1.

Schematic illustration of the synthesis process of AIGS-GH QDs.

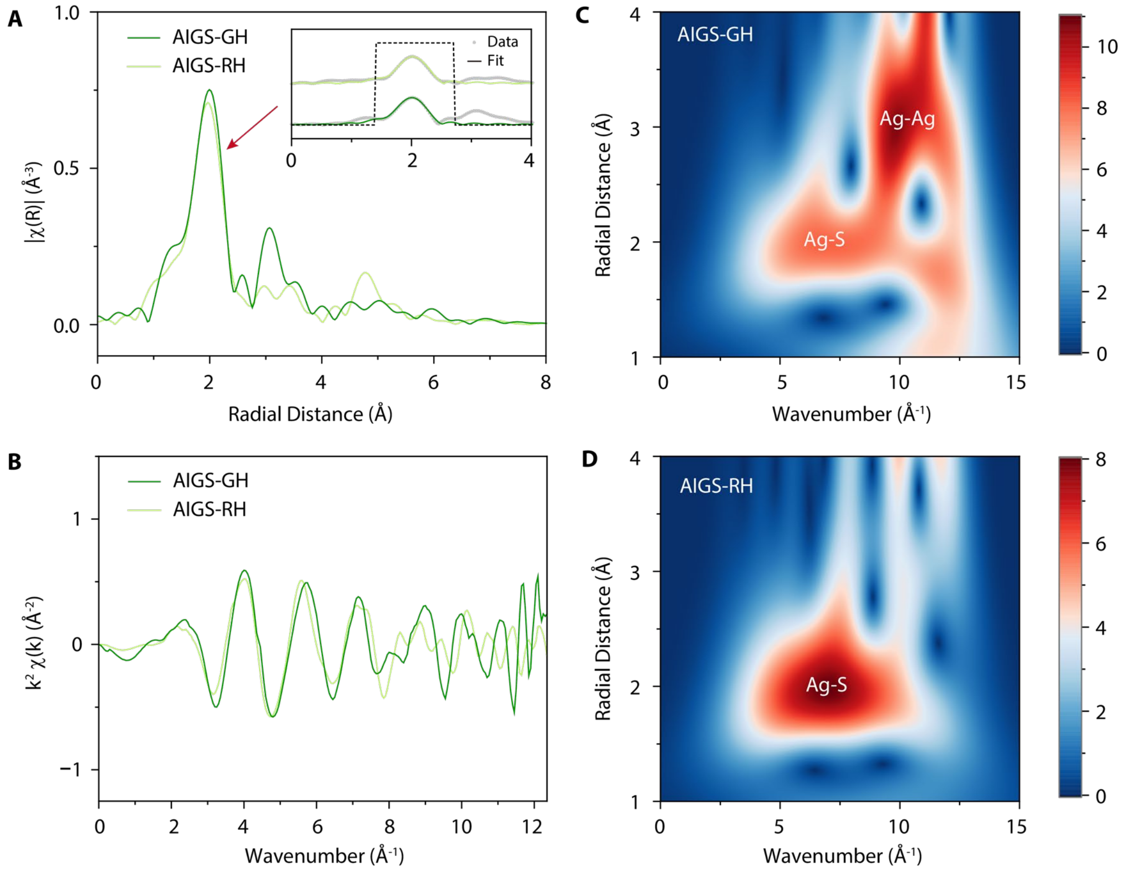

Fig. S2.

Ag K-edge EXAFS analysis of AIGS QDs. (A) Fourier-transformed R-space spectra with first-shell fitting (inset) and **(B)** k^2-weighted $\chi(k)$ spectra of AIGS-GH and AIGS-RH QDs. **(C, D)** Wavelet transform contour plots of AIGS-GH and AIGS-RH QDs.

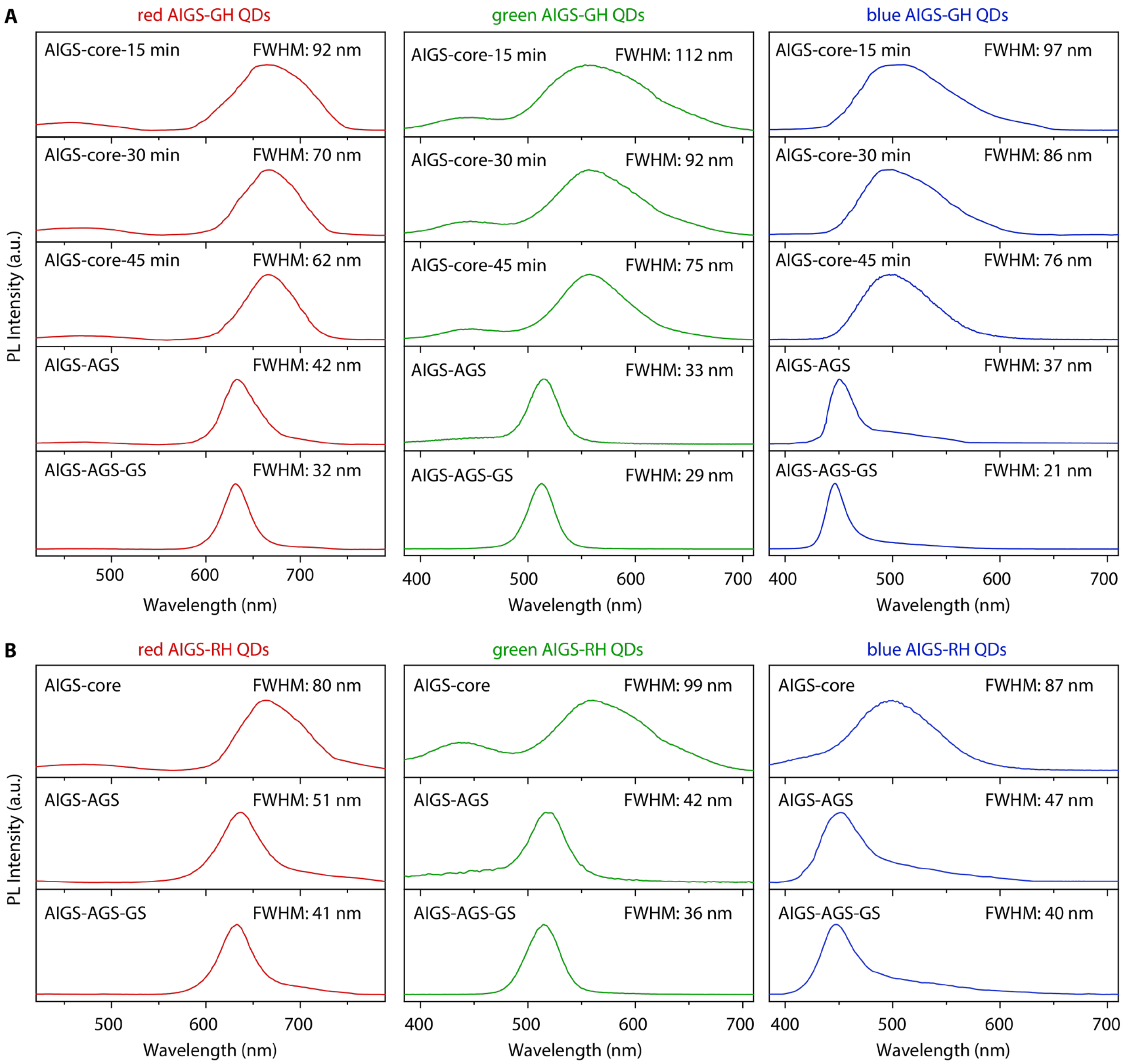

Fig. S3.

PL spectra of red, green and blue AIGS QDs at different synthesis stages. (A) AIGS-GH QDs. **(B)** AIGS-RH QDs.

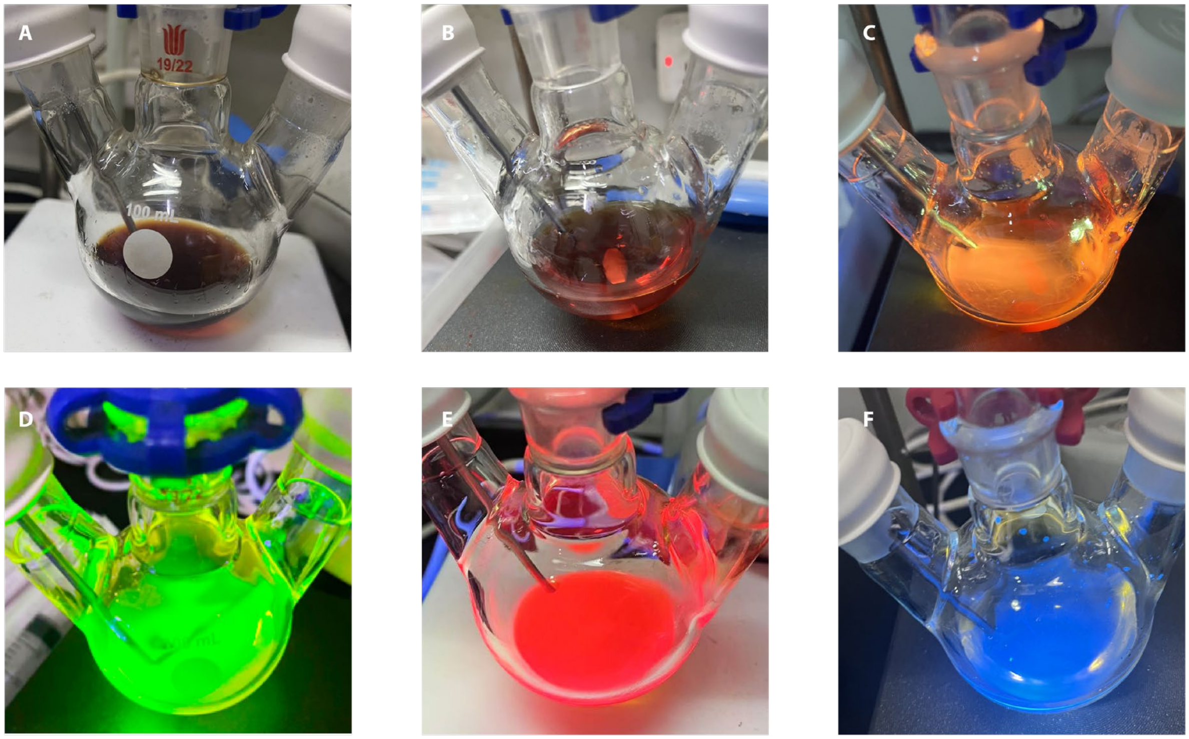

Fig. S4.

Images during the synthesis process. (**A**) AGS precursors, (**B**) AIGS cores, and photoluminescence images of (**C**) AIGS cores, (**D**) green, (**E**) red and (**F**) blue AIGS-GH QDs.

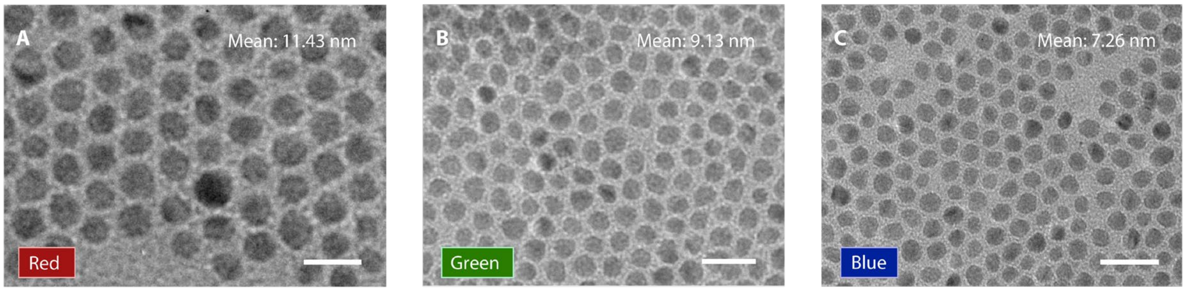

Fig. S5.

TEM images of AIGS-GH QDs. Mean radii of (**A**) red, (**B**) green, and (**C**) blue AIGS-GH QDs are 11.73 nm, 9.13 nm and 7.26 nm, respectively. (All scale bars = 20 nm)

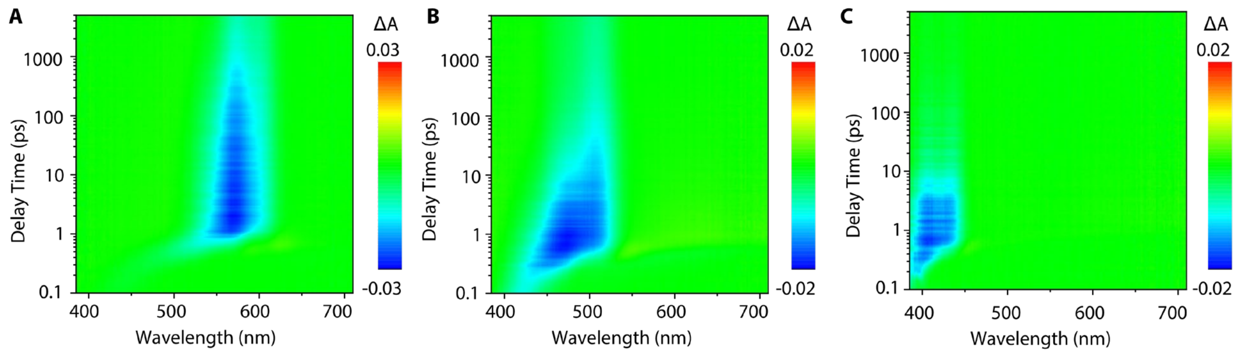

Fig. S6.

Pseudo color TA map of AIGS-AGS-GS QDs. (**A**) Red, (**B**) green and (**C**) blue AIGS-AGS-GS QDs.

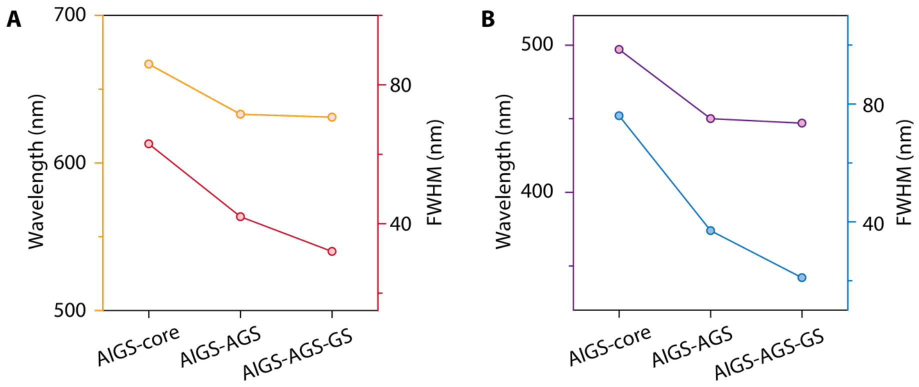

Fig. S7.

The progression of PL peak positions and FWHMs for red and blue AIGS-core, AIGS-AGS, and AIGS-AGS-GS QDs. (**A**) The red AIGS-core QDs exhibit a FWHM of 63 nm, the red AIGS-AGS QDs exhibit a FWHM of 42 nm, and the red AIGS-AGS-GS QDs exhibit a FWHM of 32 nm. (**B**) The blue AIGS-core QDs exhibit a FWHM of 76 nm, the blue AIGS-AGS QDs exhibit a FWHM of 37 nm, and the blue AIGS-AGS-GS QDs exhibit a FWHM of 21 nm.

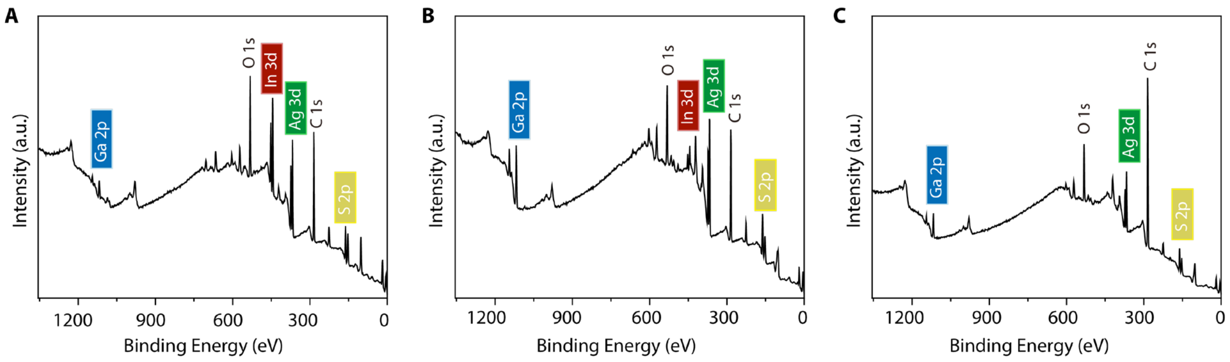

Fig. S8.

XPS spectra of AIGS-AGS-GS QDs. (**A**) Red, (**B**) green and (**C**) blue AIGS-AGS-GS QDs.

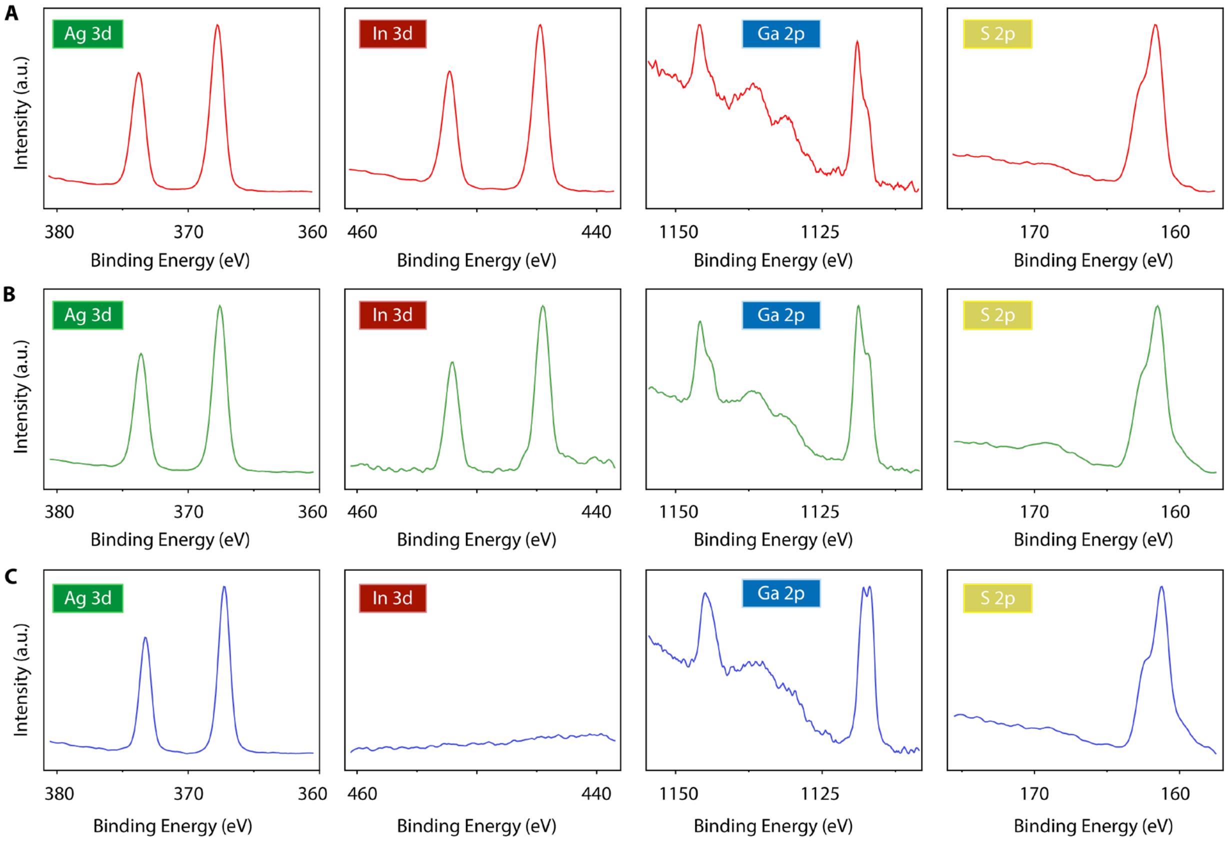

Fig. S9.

High-resolution XPS (HR-XPS) spectra of Ag 3d, In 3d, Ga 2p and S 2p for AIGS-AGS-GS QDs. (**A**) Red, (**B**) green and (**C**) blue AIGS-AGS-GS QDs.

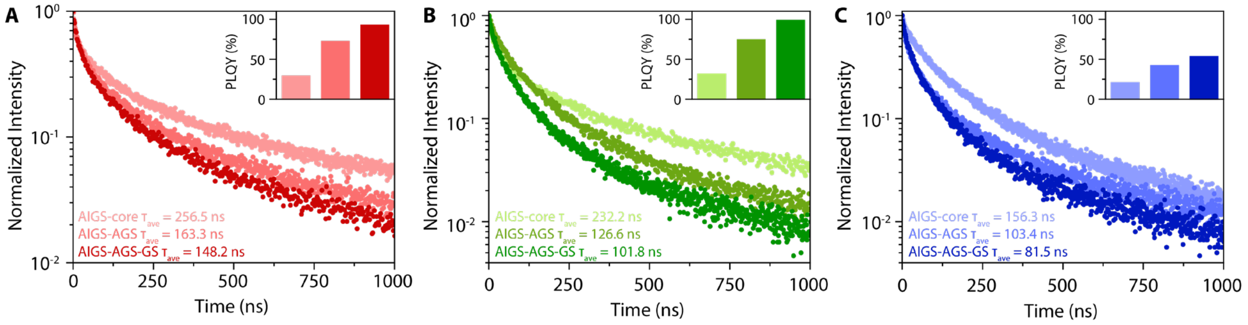

Fig. S10.

PL decay curves and PLQY of AIGS-GH QDs at different synthesis stages. The average lifetimes of (**A**) red, (**B**) green, and (**C**) blue AIGS-AGS-GS QDs are 148.2 ns, 101.8 ns, and 81.5 ns, respectively.

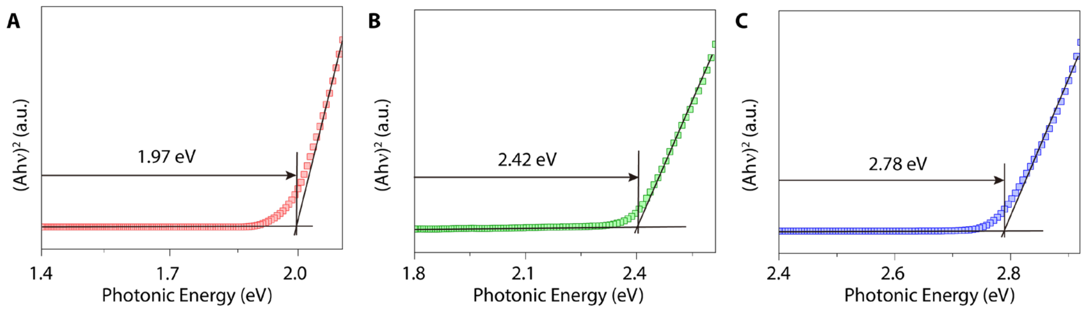

Fig. S11.

Extrapolation of the spectra to determine the bandgap energy (E_g). The bandgap energies of (**A**) red, (**B**) green, and (**C**) blue AIGS QDs are 1.97 eV, 2.42 eV, and 2.78 eV, respectively.

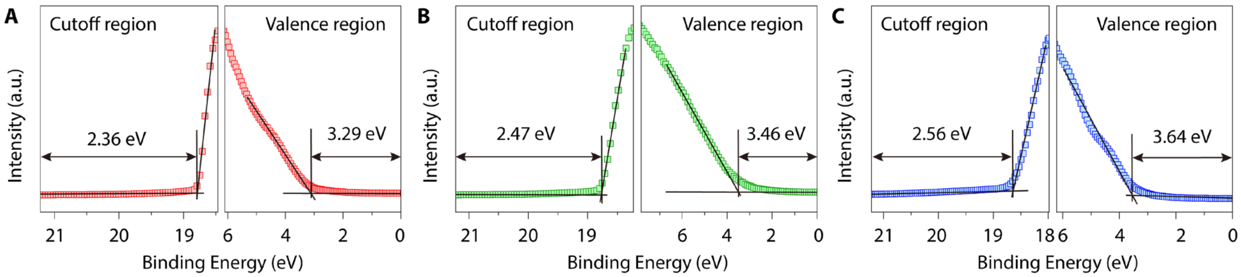

Fig. S12.

Ultraviolet photoelectron spectroscopy (UPS) measurements. The analytical results show the secondary electron cut-off and valence band edge binding energies of (**A**) red, (**B**) green, and (**C**) blue AIGS QDs.

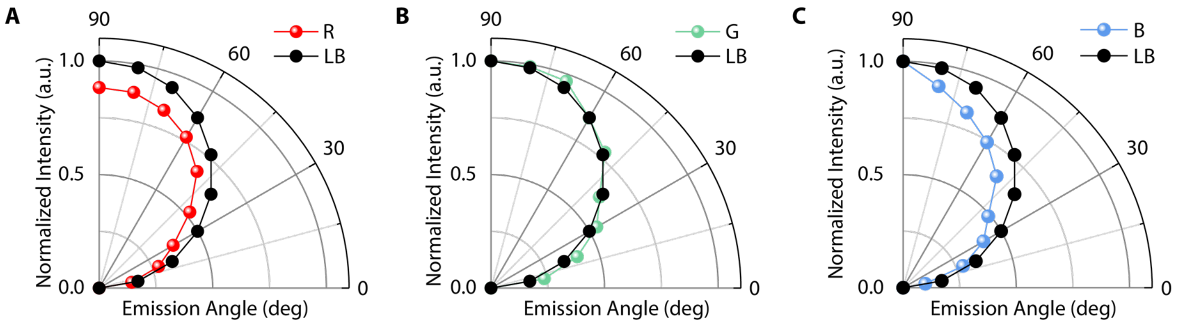

Fig. S13.

Angular luminance distributions for (A) red, (B) green and (C) blue AIGS-based QLEDs.
All the emission profiles of these devices are close to Lambertian.

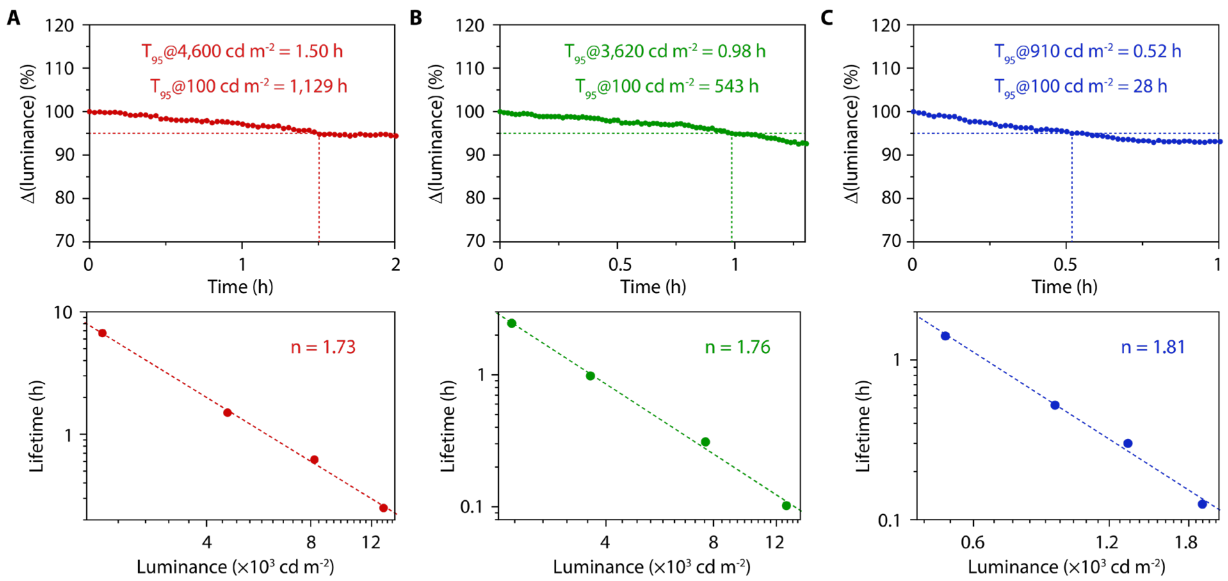

Fig. S14.

Lifetime measurements of the (A) red, (B) green and (C) blue AIGS-based QLEDs fitted with exponential curve.

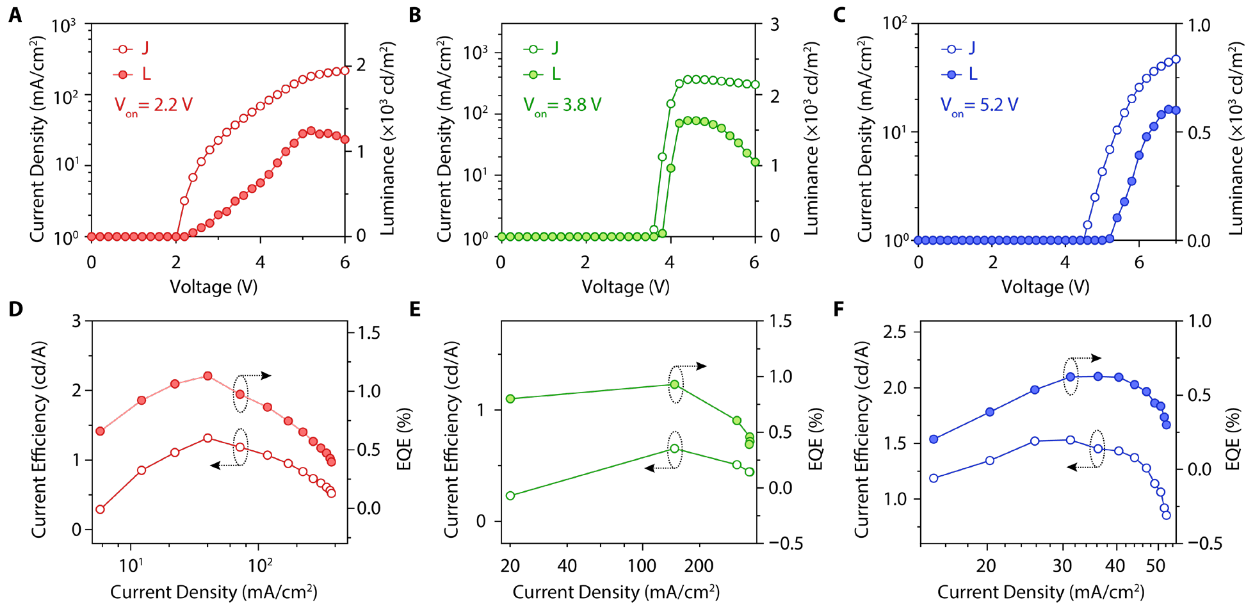

Fig. S15.

QLED performance based on AIGS-RH QDs. Current densities (hollow circles) and luminance (solid circles) versus voltage of **(A)** red, **(B)** green and **(C)** blue AIGS-RH QLEDs. Current efficiencies (hollow circles) and EQEs (solid circles) versus current densities of **(D)** red, **(E)** green and **(F)** blue AIGS-RH QLEDs.

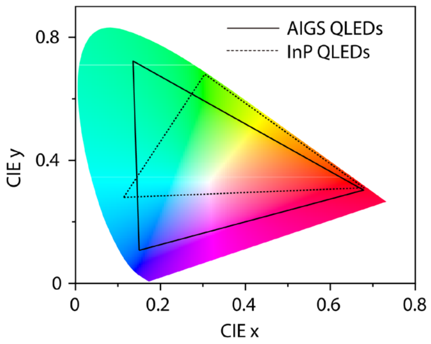

Fig. S16.

CIE color coordinates of our AIGS-based QLEDs compared with InP-based QLEDs. The AIGS-based QLEDs exhibit a broader color gamut.

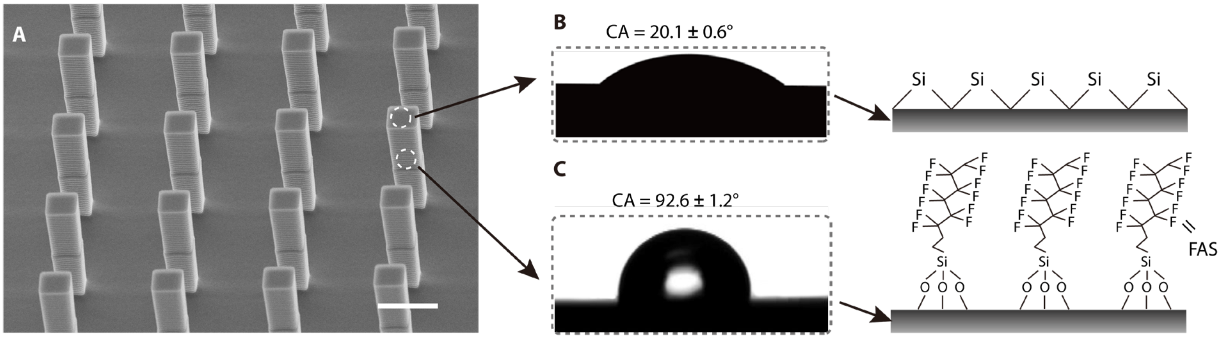

Fig. S17.

Asymmetric wettability modification of the micropillar template. (**A**) SEM image of micropillar template with asymmetric wettability modified by FAS. (**B**) The contact angle (CA) of the micropillar's top is 20.1° ± 0.6°, exhibiting hydrophilicity. (**C**) CA of the micropillar's side is 92.6° ± 1.2°, exhibiting hydrophobicity.

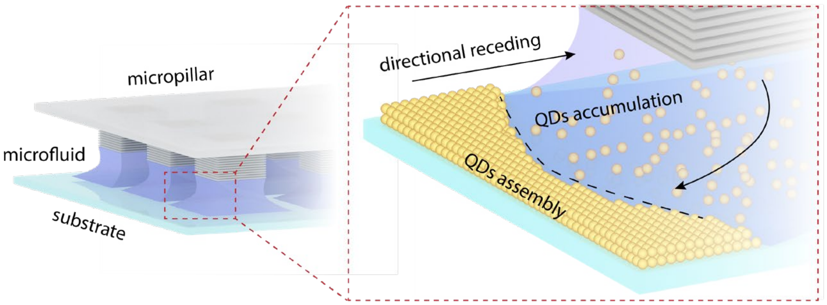

Fig. S18.

Schematic illustration of the confined assembly system to assemble QDs. Microfluids spontaneously form from the liquid film confined between the template and the substrate during solvent evaporation. As the solvent evaporated, the TPCLs on the side of the micropillar tops were pinned due to the abrupt change in wettability and geometry, forming a series of coaxial gas-liquid interfaces between the pillar tops and the substrate to confine the assembly of QDs.

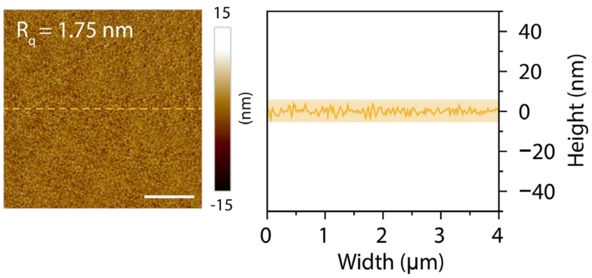

Fig. S19.

AFM surface roughness of AIGS QD microarrays. measuring 1.75 nm (scale bars = 1 μm)

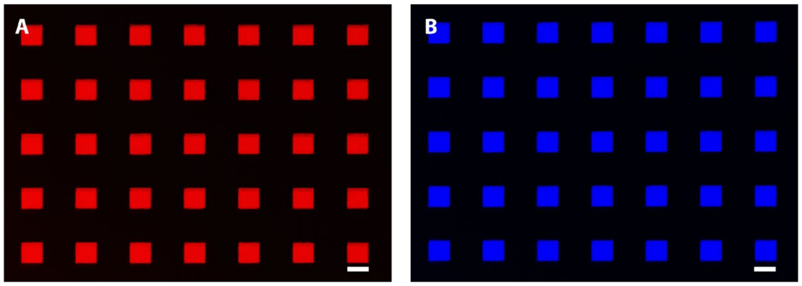

Fig. S20.

Fluorescence microscope images AIGS QDs microsquare arrays. (**A**) Red and (**B**) blue AIGS QDs microsquare arrays show high surface uniformity. (all scale bars = 20 μm)

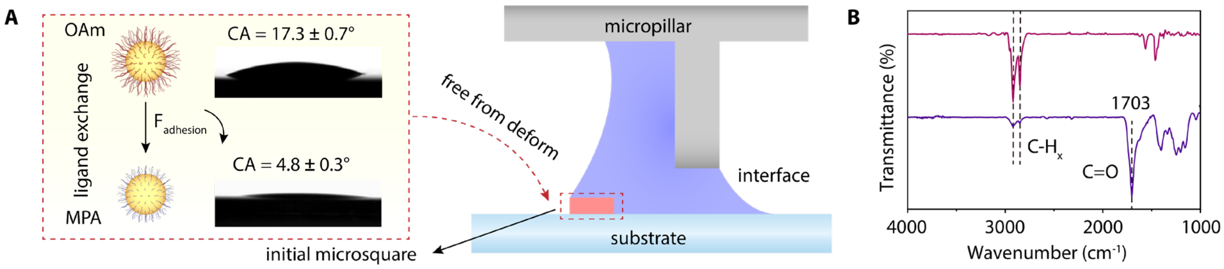

Fig. S21.

(A) Schematic illustration and contact angle measurements of the surface ligand exchange on microarrays and the hierarchical confined assembly process. (B) FTIR spectra before and after the exchange of AIGS QD ligands.

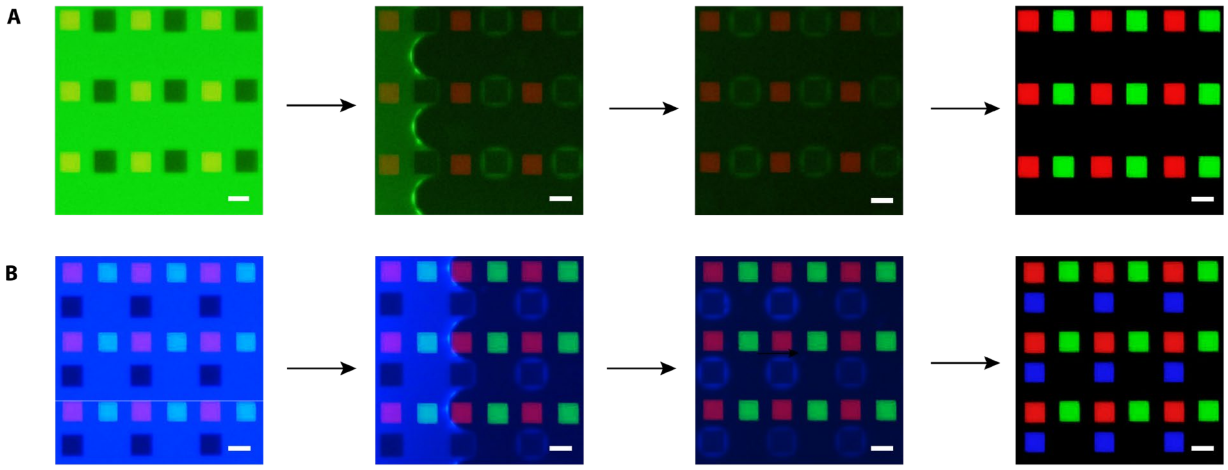

Fig. S22.

Fluorescence microscope images in different states during the assembly process of QD microarrays. (**A**) The in-situ process of assembling green QD arrays after assembling red QD arrays. (**B**) The in-situ process of assembling blue QD arrays after assembling red and green QD arrays. (all scale bars = 20 μm).

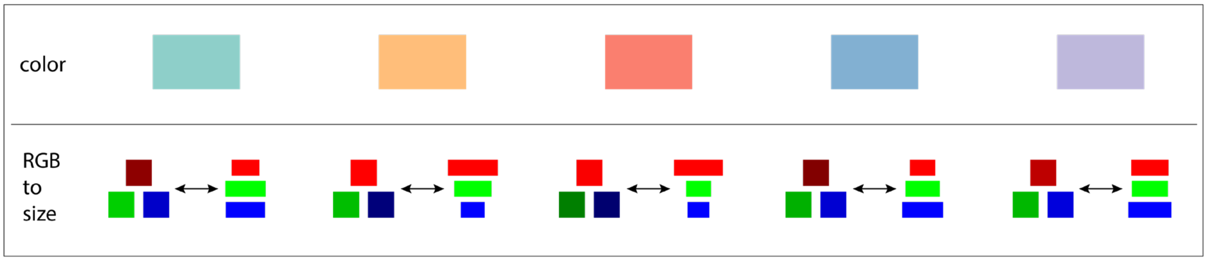

Fig. S23.

Schematic illustration of the principle for achieving externally excited full-color image. Brightness variations represented by RGB values are converted into proportional lengths of micro-rectangles. Specifically, a color with RGB values r, g, and b is represented by three micro-rectangles whose lengths are proportional to r, g, and b, respectively.

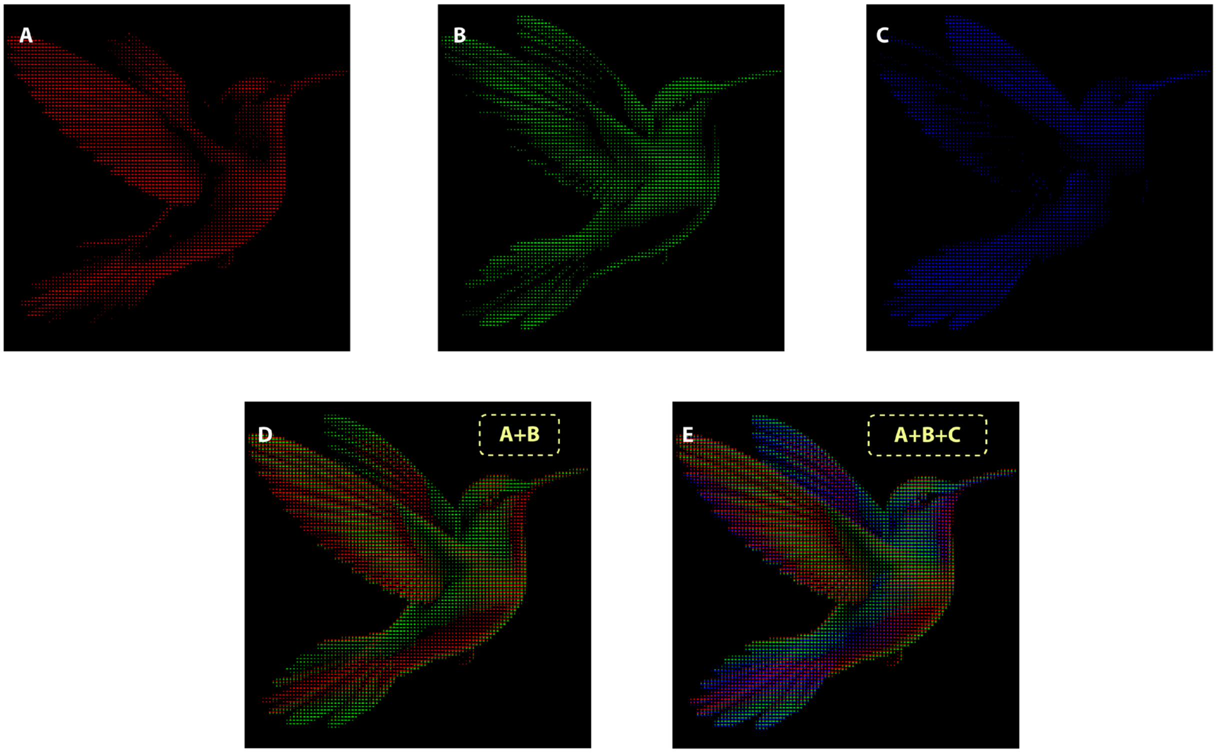

Fig. S24.

The method of realizing externally excited full-color image. By separating the RGB channels of the image and converting them into corresponding (**A**) red, (**B**) green, and (**C**) blue rectangular arrays. (**D**) Assemble the red channel first and then the green channel. (**E**) Reassemble the blue channel subsequently produces full-color image.

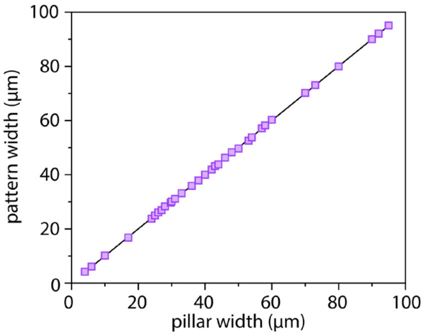

Fig. S25.

Statistical analysis of the QD array pattern width compared to the pillar width. The patterning yield of the photoluminescent full-color display is ~100% across a broad spectrum of pattern sizes.

Table S1.
Comparison of our AIGS QDs with reported Cd-free Ag-based QDs in terms of PL wavelength, FWHM and PLQY.

Color	PL (nm)	FWHM (nm)	PLQY (%)	Ref.
Red	610	42	86	(*16*)
Red	631	38	14	(*24*)
**Red**	**614**	**32**	**93**	**This work**
Green	525	34	45	(*24*)
Green	532	32	16	(*19*)
Green	528	33	95	(*30*)
Green	522	31	53	(*23*)
Green	517	30	96	(*16*)
Green	530	31	90	(*31*)
**Green**	**513**	**29**	**98**	**This work**
Blue	470	48	34	(*24*)
Blue	468	34	49	(*16*)
**Blue**	**447**	**21**	**53**	**This work**

Table S2.

Summary of GSB fitting parameters for AIGS-AGS-GS QDs synthesized via multi-step temperature control strategy.

QDs	Peak position (nm)	τ_1 (ps)	α_1 (%)	τ_2 (ps)	α_2 (%)	τ_3 (ps)	α_3 (%)	τ_{ave} (ps)
**Red**	Peak A: 574	150	36.7	5470	22.8	13000	40.5	6565
**Red**	Peak B: 599	62.1	13.1	744	29.1	51500	57.7	30090
**Green**	Peak A: 473	9.94	56.2	118	24.8	24700	18.9	4701
**Green**	Peak B: 509	83	10.6	36000	50.4	53100	39.0	22532
**Blue**	Peak A: 406	8.22	76.1	220	15.6	17200	8.22	1457
**Blue**	Peak B: 432	9.38	75.9	241	17.4	6740	6.73	503

Table S3.

Summary of PL lifetime components of AIGS-AGS-GS QDs synthesized via multi-step temperature control strategy.

QDs	τ_1 (ns)	τ_2 (ns)	τ_3 (ns)	A_1 (%)	A_2 (%)	A_3 (%)	τ_{ave} (ns)
**Red**	38.3	247	1420	69.0	27.1	3.9	148.2
**Green**	65.2	516	/	91.9	8.1	/	101.8
**Blue**	29.1	131	787	68.6	28.3	3.1	81.5

Table S4.

Comparison of our AIGS-based QLEDs with previously reported high-performance Cd-free Ag-based QLEDs in terms of peak EQE and peak luminance

QDs	Year	EL_{max} (nm)	EQE_{max} (%)	L_{max} (cd m^{-2})	Ref.
**$AgInS_2/GaS_x$**	2020	570	0.54	120.5	(*17*)
**AgGaZnS**	2022	483	0.40	123.1	(*18*)
**$AgInGaS/GaS_x$**	2023	531	1.1	175	(*19*)
**Na-AgInGaS**	2023	563	0.6	4	(*20*)
**AgCuInGaS/GaS**	2023	665	1.77	~500	(*21*)
**$AgIn_xGa_{1-x}S_2/AgGaS_2$**	2024	535	0.75	2747	(*22*)
**AgInGaS/GaS**	2024	524	5.4	~1800	(*23*)
**$AgIn_xGa_{1-x}S_2/GaS_y$**	2024	536	1.5	~70	(*32*)
**$AgInS_2/GaS_y$**	2024	586	2.0	1193	(*32*)
**AgInGaZnS**	2024	482	0.53	405	(*24*)
**AgInGaZnS**	2024	532	0.13	184	(*24*)
**AgInGaZnS**	2024	620	0.02	196	(*24*)
**AgInGaS-AgGaS-GaS**	2025	638	13.2	30670	**This work**
**AgInGaS-AgGaS-GaS**	2025	520	8.0	26450	**This work**
**AgInGaS-AgGaS-GaS**	2025	456	2.9	3580	**This work**
